# Effects and Working Mechanisms of a Multilevel Implementation Program for Applying Shared Decision-Making while Discussing Systemic Treatment in Breast Cancer

**DOI:** 10.3390/curroncol30010019

**Published:** 2022-12-23

**Authors:** Haske van Veenendaal, Loes J. Peters, Esther van Weele, Mathijs P. Hendriks, Maaike Schuurman, Ella Visserman, Carina G. J. M. Hilders, Dirk T. Ubbink

**Affiliations:** 1 Erasmus School of Health Policy & Management, Erasmus University Rotterdam, 3000 DR Rotterdam, The Netherlands; 2 Dutch Association of Oncology Patient Organizations, Godebaldkwartier 363, 3511 DT Utrecht, The Netherlands; 3 Department of Surgery, Location University of Amsterdam, Amsterdam University Medical Centers, Meibergdreef 9, 1105 AZ Amsterdam, The Netherlands; 4 Vestalia, Acaciapark 136, 1213 LD Hilversum, The Netherlands; 5 Department of Medical Oncology, Northwest Clinics, Wilhelminalaan 12, 1815 JD Alkmaar, The Netherlands; 6 Dutch Association of Breast Cancer Patients, Godebaldkwartier 363, 3511 DT Utrecht, The Netherlands; 7 Board of Directors, Reinier de Graaf Hospital, Reinier de Graafweg 5, 2625 AD Delft, The Netherlands

**Keywords:** shared decision-making, multilevel implementation, breast cancer, working mechanisms

## Abstract

Background: Enhancing the application of shared decision-making (SDM) is critical for integrating patient preferences in breast cancer treatment choices. We investigated the effect of an adapted multilevel SDM implementation program in breast cancer care. Methods: Breast cancer patients qualifying for (neo)adjuvant systemic treatment were included in a multicenter before–after study. Consultations were audio recorded between June 2018 and July 2019 and analyzed using the five-item Observing Patient Involvement in Decision-Making (OPTION-5) instrument to score SDM application by clinicians. The Shared Decision-Making Questionnaire (SDM-Q-9) was used to rate patients’ perceived SDM level. Consultation duration, decision types, number of options discussed and consultations per patient were monitored. Regression analysis was used to investigate the correlated variables and program components. Results: Mean OPTION-5 scores increased from 33.9 (*n* = 63) before implementation to 54.3 (*n* = 49) after implementation (*p* < 0.001). The SDM-Q-9 scores did not change: 91.1 (*n* = 51) at baseline versus 88.9 (*n* = 23) after implementation (*p* = 0.81). Without increasing consultation time, clinicians discussed more options after implementation. The regression analysis showed that exposure to the implementation program, redistribution of tasks and discussing feedback from consultations was associated with a higher level of SDM. Conclusion: The multilevel program helped clinicians achieve clinically relevant improvement in SDM, especially when it is tailored to (individuals in) teams and includes (e-)training, discussing feedback on consultations and redistribution of tasks.

## 1. Introduction

Breast cancer is the most common cancer in women worldwide [1]. Patients with early stage breast cancer have a (very) good long-term prognosis, with a five-year survival of 85–90% in high-income countries [2]. Chemotherapy or hormone therapy improves survival [3], but this benefit only applies to a small proportion of patients and must be weighed against the high risk of side effects. As these choices have significant implications for the patients’ quality of life and clinician preferences can strongly influence treatment decisions, it is critical to explicitly integrate patient preferences in their treatment choices [4].

Shared decision-making (SDM) is a collaborative process that integrates patient values and preferences with clinical evidence about available options and their risks and benefits, to arrive at patient-centered decisions about diagnosis, treatment or follow-up when more than one medically reasonable option is available [5,6]. Especially in Western healthcare settings, SDM is considered as an important pillar of patient-centered care and value-based healthcare [5,7], and an ethical imperative [8]. Designating concrete steps that address core elements in the SDM process that is meant to take place, e.g., step models and recognizable examples, can help raising clinician’s awareness and make them realize what SDM means for their own context [9,10,11]. 

The popularity of SDM is understandable given the positive effects: SDM-enhancing interventions such as decision aids make patients, also in low health-literacy groups, more active in decision making while making choices that better match their personal values, without adverse effects on anxiety, health outcomes or patient satisfaction [12,13,14]. Similar outcomes, such as increased patient confidence in treatment decisions, treatment satisfaction and confidence in their clinicians, are reported in oncology [15,16]. Positive experiences are also reported for clinicians, such as a lower risk of burnout [17], and encounters with breast cancer patients that are both more structured and more interesting [11]. In addition, over the years, patients themselves want to become more involved in decision-making [18]. This is especially challenging for clinicians to achieve in decisions about systemic therapy with (older) breast cancer patients, as patients might seem passive but do prefer involvement and, therefore, need explicit encouragement to participate [19].

However, determining the effectiveness of an innovation does not guarantee its inclusion in daily practice [20]. This also holds for SDM [21,22], especially in the case of (breast) cancer given its life-threatening character and the complex medical information of the many available options [23]. Therefore, implementation efforts have increased in recent years [24]. This has taught us that multilevel approaches seem necessary, using different interventions in a tailor-made approach [25,26,27]. Ultimately, implementing SDM may require that the organizational culture is transformed, and leadership and rewards focus on adopting (more) SDM as part of continuous improvement [28,29]. Direct observation of clinical encounters followed by structured feedback and coaching is educationally valuable [30] and seems promising for improving SDM behaviors [31,32,33]. Poor post-trial implementation of decision aids can be improved by incorporating them in the clinical workflow, especially by an accurate timing and an explicit report of the multidisciplinary team that treatment “is to be discussed with the patient” [34].

In a previous study on which this research builds, a multilevel implementation program appeared to improve the adoption of SDM behavior of clinicians over time, as observed during consultations regarding the surgical phase of breast cancer [25]. This program was grounded in theoretical literature and used a four-level framework for designing an effective implementation strategy [35]. In an evaluation study, clinicians especially appreciated that the program: (1) made clear how SDM was of benefit to themselves and patients, (2) contained both theory-based and practical feedback and training, (3) included a focus on the team and care pathway and (4) involved patients [11]. The feedback of the participants was used to refine the program. While it confirmed that a multi-level systematic approach is needed to achieve SDM implementation, this and other similar studies fail to provide any clues as to which parts of the program are most effective.

The primary aim of this study is to investigate whether an improved multi-level SDM implementation program, which explicitly provides patients with reflection time, helps clinicians to adopt SDM while discussing systemic treatment in breast cancer and whether patients experience more involvement in decision making. This study attempts to reveal the relative contribution of program components (working mechanisms) that lead to increased SDM application. The second study aim is to detect whether applying more SDM influences important features of consultations, such as the consultation duration, the number of options discussed and the type of decisions made. 

## 2. Materials and Methods

### 2.1. Study Design

The Standards for Reporting Implementation Studies (StaRI) was used as a guideline for the design and report on the study [36]. An unpaired before–after implementation study was used to evaluate whether a multilevel implementation program would enhance the level of SDM of clinicians observed in consultations [25]. 

### 2.2. Study Population and Selection 

Five breast cancer outpatient clinics in the Amsterdam region of the Netherlands were invited to participate in the study. An intake interview was conducted with each hospital. A hospital team was included if they were willing to invest the time required for the project and if training in SDM had not recently taken place. Both the clinical team and the research team had to be positive about collaboration. All clinicians involved in the patient’s decision-making process regarding systemic treatments for breast cancer were asked to participate in the study. Clinicians had to have the intention to participate in both the pre-test and the post-test. If a team did not accept the invitation, a team from another hospital was approached.

Patients qualified for inclusion if diagnosed with early-stage breast cancer with an indication to discuss (neo)adjuvant systemic treatment. Patients were eligible if they, or an accompanying person, spoke Dutch fluently. Each patient received information about the study and what their cooperation would entail. All patients completed an informed consent form. Each patient was included only once.

### 2.3. Ethical Approval

Approval for the study was obtained from the medical ethics review board (W16.019). 

### 2.4. Implementation Program 

The initial design of the implementation program has been described in prior publications [11,25]. It was theory-based, drew implementation lessons from previous research and paid attention to explicitly giving patients time to reflect on current choices. This theoretical framework for implementation was used to respond effectively to identified barriers to and facilitators of SDM implementation [35]. The model distinguishes four implementation levels, on which promising implementation strategies are designed: (1) Innovation: the concept of SDM; (2) Users of the innovation; (3) Organizational context; and (4) Sociopolitical context.

The program was initially tested from April 2016 to September 2017, for early-stage breast cancer (surgical) treatments among six outpatient teams of hospitals in the Utrecht region [25]. At the end of the implementation, the program was evaluated [11]. The outcomes of the evaluation of phase 1 were used to adapt the implementation program for this study (Table S2). The following changes were made to the program. (1) Feedback: the written feedback was individualized (if multiple clinicians were involved in the decision-making process with one patient) and described more concretely which communication behavior would increase the existing score. (2) Training: for improving skills, an actor was present (instead of role-play by clinicians themselves) to practice SDM in consultations based on self-reported clinical cases. (3) Patient involvement: an attempt was made to strengthen the bond with a local patient representative. (4) Project team: each hospital team was given a permanent contact person and provision of information to hospital teams, especially about planning, was improved. (5) Collaborative meetings: the number of organized collaborative team meetings, was reduced from four in the previous trajectory to two meetings during this program. This change was made because it was difficult for clinicians from different hospitals to find time in their busy schedules at the same time.

Concurrent activities that might influence the level of SDM were monitored via a logbook. A clinician from each team, together with one of the researchers, kept this logbook. Patients were involved during all phases of the program: they participated in the design, implementation and evaluation of the program as members of the research/project team. They contributed to the integration of the patient’s perspective in providing the feedback on consultations. Moreover, for each hospital team there were local patient representatives who participated in the training and were available for questions from the team.

### 2.5. Data Collection 

Each hospital team was asked to audiotape 15 decision-making processes before and after implementation of the program. One decision-making process measurement was defined as one or more consultations conducted by one or more clinicians from the team to make one decision about systemic breast cancer treatment. Patients and clinicians were aware of this and were instructed to proceed with their consultation as normal (pre-intervention) or to apply what they had learnt during the intervention (post-intervention). Recruitment of all consecutive patients newly diagnosed with breast cancer and facing a treatment decision, took place between June 2018 and October 2018 (pre-implementation), and between March 2019 and July 2019 (post-implementation).

As primary outcome measure, the OPTION-5 instrument was used to measure the extent to which clinicians involved their patients in the decision-making process during audio-recorded real-life consultations [37]. This instrument scores five key decision-making behaviors of the clinician and was found to be suitable for use in oncology [38]. Each of the five items can be rated on a scale ranging from 0 (no effort made) to 4 (exemplary effort made), leading to an overall mean score that is expressed as a percentage of the maximum score. The higher the score, the better the clinician’s SDM behavior was during the consultation. Each audiotaped consultation was independently rated by two researchers out of a team of three (HvV, EvW, GB) by using the OPTION-5 coding scheme (http://www.glynelwyn.com/observer-option-5-2014.html (accessed on 24 November 2022)). The coding scheme has been adapted for vascular surgery and oncology [39] and was refined to the specific context of discussing systemic treatment in breast cancer to increase inter-rater agreement when scoring the audio-recordings (Table S1). These independent scores of the two raters were compared and discussed to reach agreement on the final score. To determine the inter-rater agreement, unweighted Cohen’s Kappa (κ) values were calculated. Its value ranges from −1 to 1, with values greater than 0.6 considered substantial [40].

As secondary outcome measure, patients rated their perceived involvement in the decision-making process at the end of the final consultation when a decision about breast cancer treatment was made, by completing the SDM-Q-9 questionnaire [41,42,43]. Patients scored nine statements on a six-point scale, ranging from ‘completely disagree’ (0) to ‘completely agree’ (5). To calculate a percentage of the maximum score, ranging from 0 (no SDM) to 100 (maximum level of SDM), the scores of the 9 items per patient were added up and multiplied with 20/9. Questionnaires were excluded when the patient left three or more items unanswered. If one or two values were missing, these were imputed by the mean of the items that were scored [42].

Finally, factors possibly correlated with OPTION-5 scores were recorded, and for each team, the participation of each clinician in the measurements and implementation activities of the program was monitored (as part of the logbook) to explore the working mechanisms of the program in terms of the relative benefit of each part of the program. The logbook was maintained by a researcher in consultation with the contact person of each clinical team. 

To identify key features of consultations, other outcomes retrieved from the audio-recorded consultations were consultation duration, number of options being discussed and type of decisions that were made. 

### 2.6. Sample Size Calculation 

It is conceivable that relatively high OPTION-5 scores can be observed in oncology, because good communication about (breast) cancer is generally acknowledged to be important when there is a clear (shared) decision moment from the perspective of clinicians [44,45,46]. For the calculation of an adequate sample size, a mean OPTION-5 score of 39 before the intervention was used, as was found in the previous breast cancer study [25,32]. A total sample size of recordings from 120 patients (ideally 60 in the pre-measurement and 60 in the post-measurement) was needed to perform an unpaired t-test, based on an expected improvement in mean OPTION-5 score from 39 before implementation to 49 after implementation, with a standard deviation of 13, an intra-cluster correlation rho of 0.01 (to correct for interhospital differences), an α of 0.05, a power of 80% and an effect size of 0.77. To anticipate possible recording failures or dropouts, the goal was to include a total of 150 recordings of patient encounters.

A minimal improvement of 10 points was considered clinically relevant, as clinicians’ efforts in applying SDM will improve from ‘minimal’ to ‘moderate’ after an average half-point improvement on each of the five items [25].

### 2.7. Statistical Analysis 

Descriptive statistics are reported as percentages, or mean (with standard deviation; SD) or median (with interquartile range; IQR) values. Differences are presented as risk differences (RD) or mean differences (MDs) with their 95 per cent confidence intervals (95%CI). To examine differences between categorical variables, Pearson’s statistic was used. Statistical analysis was carried out using SPSS Statistics v. 28.0 (IBM, Armonk, NY, USA). P values < 0.050 (two-sided) were considered statistically significant.

After selecting the variables related to the observed SDM level through univariable regression, a backward stepwise regression analysis was performed until all remaining variables in the model had a *p*-value <0.05. These would be the independent factors significantly related to the OPTION-5 score. To give a complete picture, all variables were reported in the model. This regression analysis also corrects for the expected collinearity between variables [47]. First, variables that were not part of the intervention were analyzed: (1) clinical team, (2) type of clinician, (3) number of options discussed, (4) total exposure to program implementation, (5) consultation duration. Subsequently, the components of the implementation program were analyzed. The variables recorded in the log, on which the 5 participating teams had exactly the same results were combined in the regression model: (1) completion of E-learning and reallocation of tasks; (2) use of a decision tool, adjustment of care path and appointment of coordinator; (3) having discussed feedback from consultations; (4) patient involvement; (5) participation in training; (6) participation in collaboration meetings; and (7) number of recordings submitted.

## 3. Results

### 3.1. Participants

#### 3.1.1. Hospital Teams

Five out of a total of seven approached hospitals participated in the study. Two hospitals (one teaching hospital and one general hospital) in the Amsterdam region did not accept the invitation to participate because their doctors considered the expected time investment (12–20 hours per clinician) too high in relation to the expected benefit. Therefore, two (teaching) hospitals outside the Amsterdam region were asked to participate, both of which agreed to participate. All teams performed pre- and post-intervention decision-making process measurements. Participation in the activities of the implementation program differed per team (Table 1), e.g., one hospital only included two patients in the post-measurement.

The participating clinicians before and after the implementation were similar regarding background and education. Before implementation, 7.9% of the participants were surgeons, and after implementation, 10.4% were surgeons. For nurse specialists and medical oncologists, these percentages were 9.5% and 82.5%, respectively, before implementation, and 16.7% and 72.9%, respectively, after implementation. The practitioners who participated in the post-measurement were the same, except for one oncologist, who only participated in the pre-implementation measurement, and three nurse practitioners who were replaced between the pre- and post-implementation measurements.

There was no registration in the logbooks of concurrent activities unrelated to the program that might have influenced the level of SDM. Two hospital teams adapted the care process: in one hospital, the consultation with a nurse or nurse specialist now preceded the consultation with the medical oncologist (instead of afterwards), and in the other hospital, an additional consultation with the nurse or nurse specialist was offered in addition to the consultation with the medical oncologist.

#### 3.1.2. Patients

A total of 112 consultations of patients with newly diagnosed breast cancer were successfully recorded: 63 consultations before and 49 after implementation. Patients’ ages ranged from 25 to 86 (mean 60) years (SD: 13).

### 3.2. Characteristics of Consultations

The options discussed were chemotherapy, hormone therapy, combined therapy (chemo/targeted therapy, chemo/hormone therapy, hormone/chemo/targeted therapy) and active surveillance. Other options related to these decisions, such as the use of cooling caps during chemotherapy, psychological support and extra diagnostic procedures, were not scored with the OPTION-5, but clinicians did receive feedback if considered relevant for applying SDM. The mean number of consultations needed to decide about systemic treatment for breast cancer was 1.75 (range 1–5) consultations per patient. No significant difference was observed between the median duration of consultations before (36:00 min:sec) and after (40:00 min:sec) the intervention (*p* = 0.74).

In total, 77.5% of the consultations were held by clinicians who participated in feedback meetings, 38.8% were held by clinicians who participated in group meetings, 98% were held by clinicians who attended a SDM training and 30.6% were held by clinicians who completed the E-learning. All but one hospital team involved a patient representative at a local level.

### 3.3. SDM Adoption by Clinicians

All five teams showed higher total OPTION-5 scores after the implementation, although the variation among teams was considerable (Table 2). The total mean OPTION-5 scores increased from 33.9 (SD 14.8) at baseline (63 patients) to 54.3 (SD 19.9) (52 patients) after implementation (MD 20.4 (*p* < 0.001, 95% CI: 13.6 to 27.2)). The three raters reached acceptable levels of inter-rater agreement over the rated consultations (κ = 0.57, κ = 0.47 and κ = 0.60).

### 3.4. Perception of Patients 

Of the 112 included patients, 74 completed the SDM-Q-9: 51 questionnaires before and 23 after implementation (overall response rate 66.1%). Three questionnaires had to be imputed because patients left one or two items unanswered. The perceived involvement in decision-making was generally high and was not changed by the intervention: before the implementation the median score was 91.1 (IQR: 82.2−100.0) versus 88.9 (IQR:82.2−100.0) after implementation (*p* = 0.81) (Table 2). 

### 3.5. Correlated Variables 

Table 3 shows that the exposure to the implementation program and the number of discussed options with the patients are significantly correlated to the observed level of SDM among clinicians. The Beta coefficient represents the increase in OPTION-5 score of this variable compared to the reference variable: e.g., with ’high exposure’ means that the average OPTION score increases 19.6 points when the implementation program is (almost) fully implemented. No correlation was found for the clinical team, type of clinician and consultation duration.

### 3.6. Program Components (Working Mechanisms)

When analyzing the different components of the intervention program, (1) the completion of the e-learning and reallocation of tasks and (2) having discussed feedback from consultations were significantly correlated (*p* < 0.05) with the level of SDM (Table 4). No correlation was found for the use of a decision tool, adjustment of the care pathway, and appointment of a coordinator. The variables (1) patient involvement; (2) participation in training; (3) participation in collaboration meetings; and (4) number of recordings submitted, were removed due to collinearity.

### 3.7. Key Consultation Features: Duration, Discussed Options and Decisions Made

After implementation, clinicians were significantly more likely to offer four different treatment options during the consultation than before, while there was no increase in consultation duration (Table 2). Additionally, chemo/hormone therapy was discussed significantly more often after the intervention. The type of decisions did not differ significantly between the pre- and post-intervention teams (*p* = 0.41).

## 4. Discussion

### 4.1. Discussion of Results

After evaluation and adaptation, the multilevel implementation program again led to improved patient involvement in the decision-making process [11], without a significant increase in consultation duration. No effect was demonstrated on the patients’ perceived involvement in decision making as measured by the SDMQ-9. Although implementation efforts in the field of SDM are increasing [24], these results are both promising and generalizable to other (cancer) settings: The 20-point increase in observed SDM behavior, focusing on discussing systemic therapy in early-stage breast cancer, was relatively high [25,32]. Moreover, it occurred in five different clinical teams among team members from different clinical backgrounds (medical oncologists, oncology surgeons, nurses, nurse specialists). It implies that focusing the assessments on the interprofessional team performance, rather than the individual performance of each clinician, is meaningful because the possible improvements are then also approached as a team—or even organizational—performance. This is in line with the plea for addressing organizational characteristics as part of implementation approaches [27]. Therefore, it seems prudent to continue with systematic implementation approaches that focus on the team to strengthen the social support. At the same time, these approaches should allow customization for the different teams and even individual team members, especially to stimulate the intrinsic motivation needed for sustainable behavioral change [11,29,48]. The result from this research seems to recommend periodic individual feedback on each individual clinician’s consultations, in addition to interventions aimed at the team (feedback on consultations on general issues for team learning; effective division of tasks; and facilitation with, for example, decision tools and outcome data) and the organization (process redesign). Future research may focus on how to make multi-level implementation efforts more effective and easier to scale-up and may produce improvements that last, especially as part of a continuous improvement process.

Exposure to the implementation program—even moderate exposure—was found to be the most strongly correlated variable for increasing observed SDM behavior. Encouragingly, this influence is much greater than other hard-to-change variables such as the type of doctor or the length of the consultation. Within this implementation program, the finding that the redistribution of tasks, e-training, and the discussion of feedback from consultations is associated with more application of SDM, is a breakthrough. These can be added to already proven interventions such as decision aids and training [22,31,46]. Further research is needed into the possible contribution of care pathway redesign and patient involvement, also because these variables may be less related to SDM scores as such, but rather to aspects such as consultation duration, number of consultations, and patient and practitioner satisfaction. This is especially important as both patients (representatives) and clinicians in the project indicated that having time to make decisions is an important condition for participation in SDM [25,49].

The intervention helped clinicians to discuss more options with their patients, particularly combined chemo/hormone therapy. Moreover, a trend was seen towards offering active surveillance and chemotherapy more often. Previous research also indicates that patients who use a decision aid or receive more SDM more often choose the option of active surveillance [50,51]. As there seems to be shift towards choices that are more in line with the values of patients after the use of decision aids [12], this underlines the importance of SDM implementation.

The COVID pandemic has accelerated the use of hybrid care. Phone consultations were already part of our program so there seems no impediment to promoting the adoption of SDM also during digital consultations. An additional advantage of hybrid care is that it becomes easier to add an extra (digital) consultation in the care pathway to offer patients more reflection time. In addition, it may lower the threshold for patients to use digital means of communication and support, such as video information and better use of the electronic patient record. The implementation strategies, including financial compensation, will have to be adapted accordingly.

### 4.2. Strengths and Limitations 

A strength of this study was the active participation of clinicians from different backgrounds, and patient representatives in designing, implementing and evaluating the program, based on a theoretical implementation framework. In addition, due to the cooperation with the contact person of each clinical team, an adequate registration of participation in the parts of the implementation program could be obtained.

A study limitation is the before-after design without control group, especially since the program lasted more than a year. Therefore, an effort has been made to keep accurate records of the simultaneous actions taken by teams. The fact that the patients in the pre-test were different from those in the post-test also makes it uncertain whether completely comparable groups were included, while correction for disease stage and recurrence risk for instance was not possible as these data were not collected in the study. The alternative of cluster randomization was rejected because of the much higher cost in relation to the effect as well as the possibility to scale up such an implementation program. As the investigators had to provide relevant feedback as part of the implementation, patients, clinicians and investigators could not be blinded to the intervention and the recordings. Clinicians may have gone the extra mile to incorporate SDM in their consultations. However, previous research shows that the effect of this on the SDM-scores is limited [52,53]. In addition, raters may have been biased in scoring the consultations, as they knew that they were listening to pre-implementation or post-implementation recordings.

The required sample size was calculated for a 10-point increase in the primary outcome measure. This may mean that the size of this sample is insufficient to demonstrate significant effects of the secondary outcomes.

A limitation of the regression analysis was that some variables recorded in the logbooks for which the five participating teams had exactly the same results had to be merged in the model. Consequently, it is not clear which of the two variables ‘e-learning’ and/or ‘reallocation of tasks’ were significantly correlated. Finally, the pre-implementation scores were relatively high [32,54]. This was expected, as for breast cancer communication efforts are already relatively intensive as compared to other conditions [46], and the assessments were focused on the performance of the whole team. However, this may imply that for lower-scoring clinicians, the approach needs to be adjusted in some aspects. The lack of an obligation to offer an explicitly patient-oriented intervention as part of the implementation program (decision aid, three good questions, etc.) could also increase the effect of the program [12], because SDM involves the cooperation of two parties involved [55]. This was urged in the collaborative meetings and through the contact person of the teams, but the commitment to using a decision tool could not be enforced. It is worth considering to add this as a condition for participation in a program. 

## 5. Conclusions

A theory-based multilevel SDM implementation program, co-designed with patients and clinicians, was found to result in a significant and clinically relevant improvement in SDM behavior. Although it requires a reasonable (time) investment from clinicians, the supporting research team, and patient representatives, it is a temporary investment with no adverse effects such as increased consultation time in the long-term. Multilevel, theory-based approaches that can be tailored to both the challenges of the teams as a whole and individual clinicians seem preferable. Factors promoting the effectiveness of an implementation program include (e-)training, discussing feedback on consultations and redistribution of tasks in the care pathway.

## Figures and Tables

**Table 1:** Participation of hospital teams in the program.

	Participation in Team Training	Care Pathway Redesign, Decision Tool Used, Coordinator Appointed	Reallocation of Tasks/ E-Learning	Participation Clinicians in 2 Collaborative Meetings (*N*)	Patients Involved	Discussed Feedback from Consultations (*N* before; *N* after)
Team 1	Yes	Yes	No	Yes (2)	Yes	Yes (14;15)
Team 2	Yes	No	No	No (0)	No	Yes (9;9)
Team 3	Yes	No	No	Yes (4)	Yes	Yes (9;2)
Team 4	Yes	Yes	Yes	Yes (3)	Yes	Yes (16;15)
Team 5	Yes	No	No	Yes (2)	Yes	Yes (15;8)

**Table 2:** Consultation characteristics before and after the implementation program.

	Pre- Implementation	Post- Implementation	Difference (*p*-Value) (95%CI)
1. Option-5 scores (SD) (*N*)			
Hospital team 1	26.4 (11.0) (14)	58.0 (17.0) (15)	+31.6 (<0.001) (20.6 to 42.6)
Hospital team 2	28.9 (10.8) (9)	50.0 (12.2 (9)	+21.1 (<0.001) (9.6 to 32.7)
Hospital team 3	45.6 (13.8) (9)	52.5 (3.5) (2)	+6.9 (0.51) (−16.1 to 30.0)
Hospital team 4	43.8 (13.1) (16)	65.7 (21.4) (15)	+21.9 (0.002) (9.0 to 34.8)
Hospital team 5	26.3 (12.9) (15)	31.3 (11.3) (8)	+5.0 (0.37) (−6.3 to 16.2)
Total (*N* = 112)	33.9 (14.8) (63)	54.3 (19.9) (49)	+20.4 (<.001) (13.6 to 27.2)
2. Total SDM-Q-9 scores			
Median (IQR) (*N* = 74)	91.1 (82.2−100.0) (51)	88.9 (82.2−100.0) (23)	−2.2 (0.81)
3. Consultation duration			
Median min:sec (IQR) (*N*)	36:00 (24.0−70.0) (63)	40:00 (25.0–77.0) (49)	+04:00 (0.74)
4. Number of consultations			
1 per patient (%)	31 (49.2%)	24 (49.0%)	−0.2% (.98) (−17.8 to 18.2)
>1 per patient (%)	32 (50.8%)	25 (51.0%)	+0.2% (.98) (−17.8 to 18.2)
5. *N* of options offered			
1 option (%)	23.3% (14)	10.2% (5)	−13.1%
2 options (%)	60.0% (36)	53.1% (26)	−6.9%
3 options (%)	15.0% (9)	6.1% (3)	−8.9%
4 options (%)	1.7% (1)	30.6% (15)	+28.9%
Total mean	1.95% (60)	2.57% (49)	+0.62% (*p* < 0.001) (0.28 to 0.96)
6. Type of option offered			
Active surveillance	68.3% (41)	81.6% (40)	+13.3% (0.11) (−3.3 to 28.4)
Chemotherapy	50.0% (30)	67.3% (33)	+17.3% (0.07) (−1.2 to 34.1)
Hormone therapy	61.7% (37)	59.2% (29)	−2.5% (0.79) (−20.4 to 15.4)
Chemo/targeted therapy	11.7% (7)	12.2% (6)	+0.5% (0.93) (−11.8 to 14.0)
Chemo/hormone therapy	1.7% (1)	32.7% (16)	+31% (*p* < 0.001) (17.5 to 45.0)
Hormone/chemo/targeted therapy	1.7% (1)	4.1% (2)	+2.4% (0.44) (−5.4 to 12.1)
No decision yet	1.7% (1)	0.0% (0)	−1.7% (0.36) (−8.9 to 5.7)
7. Chosen options			
Conservative treatment	9.8% (5)	8.2% (4)	−1.6% (0.77) (−13.9 to 10.7)
Chemotherapy	29.4% (15)	28.6% (14)	−0.8% (0.93) (−18.2 to 16.7)
Hormone therapy	39.2% (20)	30.6% (15)	−8.6% (0.37) (−26.2 to 9.9)
Chemo/targeted therapy	13.7% (7)	8.2% (4)	−5.5% (0.37) (−18.5 to 7.5)
Chemo/hormone therapy	3.9% (2)	14,3% (7)	+10.4% (0.07) (−1.4 to 23.1)
Hormone/chemo/targeted therapy	0.0% (0)	2.0% (1)	+2.0% (0.31) (−5.2 to 10.7)
No decision yet	3.9% (2)	8.2% (4)	+4.3% (0.37) (−6.3 to 15.6)

Percentages may not add up to 100% due to rounding.

**Table 3:** Regression analysis of factors that are not part of the implementation program and exposure to implementation program.

	β-Coefficient * (95% CI)	*p*-Value
Independent variables		
1. Hospital team		
Team 1	reference	
Team 2	−4.2 (−16.8 to 8.4)	0.51
Team 3	7.7 (−4.0 to 19.3)	0.19
Team 4	−4.0 (−18.0 to 9.9)	0.57
Team 5	−10.9 (−21.8 to 0.12)	0.053
2. Type of clinician		
Medical oncologist	reference	
Nurse specialist	10.2 (−6.7 to 27,1)	0.24
Oncology surgeon	10.4 (−6.3 to 27.0)	0.22
3. Number of discussed options		
1 option	reference	
2 options	10.2 (2.7 to 17.6)	0.008
>2 options	14.4 (4.2 to 24.6)	0.006
4. Consultation duration		
<25 minutes	reference	
25–45 minutes	3.3 (−5.3 to 11.8)	0.45
>45 minutes	6.5 (−3.7 to 16.7)	0.21
5. Exposure to implementation program		
No exposure (0 activities)	reference	
Median exposure (1–5 activities)	13.1 (4.9 to 21.3)	0.002
High exposure (6–10 activities)	19.6 (11.9 to 27.3)	<0.001

This model explained 50% (Adjusted R^2^) of the variance. * For interpretation, the Beta coefficient represents the increase in OPTION-5 score of this variable compared to the reference variable.

**Table 4:** Regression analysis of components of the implementation program.

	β-Coefficient * (95% CI)	*p*-Value
Independent variables		
1. Completion E-learning and reallocation of tasks		
Not carried out	reference	
Carried out	11.4 (0.31 to 22.5)	0.044
2. Use of decision tool, adjustment of care pathway and appointment of a coordinator		
Not carried out	reference	
Carried out	6.0 (−5.3 to 17.3)	0.30
3. Having discussed feedback from consultations		
No participation	reference	
Participation	18.7 (10.0 to 27.4)	<0.001

This model explained 50% (Adjusted R^2^) of the variance. * For interpretation, the Beta coefficient represents the increase in OPTION-5 score of this variable compared to the reference variable.

## Data Availability

De-identified data can be requested from the corresponding author at haskevanveenendaal@gmail.com.

## Supplementary Materials

The following supporting information can be downloaded at https://www.mdpi.com/article/10.3390/curroncol30010019/s1. Table S1. Refined scoring definitions for the OPTION-5 manual. Table S2. Content of the multilevel implementation program using a 4-level framework for designing an effective implementation strategy [35].
