# Supplementary material for: Effects and Working Mechanisms of a Multilevel Implementation Program for Applying Shared Decision-Making while Discussing Systemic Treatment in Breast Cancer
Source: Curr Oncol. 2022 Dec 23;30(1):236–49. doi: 10.3390/curroncol30010019 (PMC9857756; doi:10.3390/curroncol30010019)
Supplement: Supplementary file 1 [file curroncol-30-00019-s001.zip › curroncol-2088632-supplementary.pdf]

Current Oncology MDPI

Supplementary materials:

Supplementary Table S1. Refined scoring definitions for the OPTION-5 manual.

Item	Description	Specification
1	The provider draws attention to - or re-affirms - a problem where alternate treatment or management options exist, and which requires the initiation of a decision- making process. If the patient draws attention to the availability of options, and the provider responds by agreeing that the options need consideration, the item can also be scored positively.	0—not observed<br>1—problem definition<br>2—listing the options<br>3—equality of the options<br>4—is it clear/any questions
2	The provider reassures the patient - or re-affirms - that he/she will support the patient in becoming better informed. The provider will support/explain the need to deliberate about the different options.	0—not observed<br>1—decide together<br>2—mention is it a difficult choice<br>3—will support irrespective of the choice of the patient<br>4—both options are OK, depends on the preferences of the patient, provider has a supportive role
3	The provider gives information - or re-affirms/checks understanding - about options that are considered reasonable (including taking 'no action'), and supports the patient in understanding/comparing the pros and cons.	0—no information<br>1—listing the options<br>2—explaining pros and cons<br>3—is it clear/any questions<br>4—ask the patient to repeat the information
4	The provider supports the patient to examine, voice, and explore his/her personal preference in response to the options that have been described.	0—not observed<br>1—exploring preferences<br>2—exploring concerns<br>3—exploring expectations<br>4—integrates preferences/ concerns/expectations for recommendation
5	The provider makes an effort to integrate the patient's preferences in terms of decisions that are either made by the patient or arrived at by a process of collaboration and discussion.	0—not observed<br>1—indicates need for decision<br>2—additional information to review the decision at home<br>3—appointment for evaluating the decision<br>4—provider indicates that the patient can abandon earlier choice
Total score 0–20; Rescale 0–100		

Supplementary Table S2. Content of the multilevel implementation program using a 4-level framework for designing an effective implementation strategy [34].

Level of implementation	Working mechanism	Form	Examples (indicative)
1. The innovation (the concept of SDM)	What means SDM and what behavior is effective during a breast cancer consultation	a. Clinicians were explained a clear practical 4-step model for applying SDM and mirror the model to their current communication behavior during consultations (sense making).<br>b. Background information about the theory of SDM, presentations of the working sessions, etc. was online available for the teams.	a. We asked teams to use the 4 SDM-steps for reflection during all program activities. We discussed and gave feedback on discussing all options (incl. surveillance), discussed and explained when and how to use teach back, we suggested to ask patients 'touching questions' to discuss what matters most to them.<br>b. We shared information about why SDM is important, about how process redesign works, relevant publications, etc.
	Supporting the use of effective decision tools to support SDM in daily practice	a. Teams were provided with an overview of tools made available to via a website that can enhance SDM, especially in relation to breast cancer systemic therapy.	a. We asked each team to experiment with a decision aid, hospitals changed their invitation letter to patients motivating them to share in the decisions they face ('Ask-the-3-questions'), hospitals rearranged their patient information map, we provided handy cards with the 4 SDM-steps, etc.
2. User of the innovation (patient and clinician)	Providing individual and team feedback on actual SDM behavior (before & after implementation)	a. Each team collected audio-recordings from 15 patients pre-intervention and 15 patients post-intervention. Feedback was provided on the performance regarding SDM and timeout in a team meeting (with recorded audio fragments from team members) and via a report containing feedback for the team as a whole and individual feedback.	a. We selected individualized quotes per clinician that illustrate the 4 steps for SDM. Characteristic audio fragments were selected and listened with teams as examples of ways to discuss issues that enhance the process of SDM, such as how to discuss risks, how to ask open questions, how to use teach back, how to explain that there's time to consider the decision etc. In addition, highlights of the team feedback report were discussed
	Training SDM via team training & e-learning	a. An inter-professional team training session addressed the application of SDM and timeout in consultations,. The training session was tailored to the needs of the team and the results of the pre-intervention measurement and included actor role-play.<br>b. An e-learning explained the theory of SDM and outlined how to apply the 4-step model in practice.	a. Standard agenda for the (2-3 hours) training: (1) discussing applicability of SDM theory and 4 steps for SDM (2) discussing personal learning questions and ways to apply SDM in a consultation (3) actor role play (4) reflection on care path and process of decision making, need for timeouts.<br>b. Content e-learning (45 minutes): definition, legal & ethical background, patient perspectives on SDM, effectiveness, when to apply SDM, how to apply SDM, pitfalls for and myths about SDM and a video example.
	Peer learning via collaborative working sessions for teams	a. Two or more team members were asked to participate in a total of 2 collaborative working sessions designed to facilitate process redesign, the application of SDM, timeout, and the selection of tools for SDM.	a. The first session took place at the start of the project, one session 2 months before the start of the post-measurement. Topics: integrating 4 steps for SDM in the care path, the use of tools and providing information to read at home, patient perspectives on time needed for SDM.
3. Organizational context	Redesign pathway including SDM and timeouts, using	a. Ask and support teams to include time-out & decision-tools in pathway. The Plan-do-check-act cycle was explained to teams and teams were encouraged to experiment	b. First consultation: explain the process of SDM, discussing treatment options and their pros and cons and ask patients what matters to them. Next, patients receive in-

	rapid cycle improvement	with new ways of working that better provide room for SDM and timeout.	formation to read at home. Second consultation: discussing preferences. Patients are encouraged to take extra time for the decision or - when they state to be ready - a decision is made.
	Reconsider working process in relation to SDM	a. Ask and support teams to adapt multidisciplinary team consultation & reassign communication tasks. .	a. Nurse (specialist) and physicians aligned their consultations, so that the 4- steps of SDM are better connected throughout the decision making process.
4. Socio-political context	Facilitating a context that supports the implementation of SDM in daily practice	a. Identify barriers and facilitators for implementation in the hospital and discuss ways to respond to them at the start of the project with the team coordinator.<br>b. Two team members per hospital were interviewed about experienced barriers and facilitators of the implementation program. This information was fed back to the teams.<br>c. Obtain commitment for time investment at the start.	a. Discussed at the start: Availability of personnel, SDM tools and patient information, room for timeout in the care path, time for the team to meet, management support, and promising actions for SDM implementation.<br>b. Perceived facilitators: making SDM specific for what is discussed in a consultation, effect on patient, individual feedback on consultations and training.<br>Perceived barriers: Team time, skills for specific patients (i.e. low health literacy).<br>c. Project plan and time investment was explained and written commitment was demanded before the start of the project.
	Access to implementation expertise on request	a. Offer expertise in the implementation of SDM, timeout, and quality improvement via a appointed permanent contact person per team.	a. Telephonic and face-to-face contact regarding questions or requests like an extra team meeting, was provided on request of the hospital team by the project team or experts.
5. Added to the levels: patient involvement	Partnering with patients and collecting patients' perspectives on SDM and timeout	a. Ensure that patient representation was systematically embedded, also at the local hospital level.<br>b. Teams were provided with the views of (ex-)breast cancer patients on preferences regarding chemo- and hormonal therapy.<br>c. Each team collected the questionnaires (SDM-Q9) of 15 patients pre-intervention and 15 patients post-intervention..	a. In the project-team, the collaborative working sessions, local team sessions and all other meetings, patient advocates and/or professional patient representatives participated.<br>b. Via a website survey 564 patients responded: looking back to the decision making process, a majority wanted to have taken more time then than they felt at the time of diagnosis. Two-third of the patients wanted to make the decision in 3 consultations with 4-7 days in between them.<br>c. The results of the questionnaire were fed back to the team via a report. Patients were generally very positive.
